# Ligand-Receptor Interactions of Galectin-9 and VISTA Suppress Human T Lymphocyte Cytotoxic Activity

**DOI:** 10.3389/fimmu.2020.580557

**Published:** 2020-11-20

**Authors:** Inna M. Yasinska, N. Helge Meyer, Stephanie Schlichtner, Rohanah Hussain, Giuliano Siligardi, Maxwell Casely-Hayford, Walter Fiedler, Jasmin Wellbrock, Cloe Desmet, Luigi Calzolai, Luca Varani, Steffen M. Berger, Ulrike Raap, Bernhard F. Gibbs, Elizaveta Fasler-Kan, Vadim V. Sumbayev

**Affiliations:** ^1^ Medway School of Pharmacy, Universities of Kent and Greenwich, Chatham Maritime, United Kingdom; ^2^ Division of Experimental Allergology and Immunodermatology, University of Oldenburg, Oldenburg, Germany; ^3^ Beamline 23, Diamond Light Source, Didcot, United Kingdom; ^4^ Department of Oncology, Hematology and Bone Marrow Transplantation with Section Pneumology, Hubertus Wald University Cancer Center, University Medical Center Hamburg-Eppendorf, Hamburg, Germany; ^5^ European Commission Joint Research Centre, Ispra, Italy; ^6^ Institute for Research in Biomedicine, Universita’ della Svizzera Italiana (USI), Bellinzona, Switzerland; ^7^ Department of Pediatric Surgery, Department of Biomedical Research, Children's Hospital, Inselspital, University of Bern, Bern, Switzerland; ^8^ Department of Biomedicine, University Hospital Basel and University of Basel, Basel, Switzerland

**Keywords:** Galectin-9, VISTA, T cells, NK cells, acute myeloid leukemia, immune escape

## Abstract

Acute myeloid leukemia (AML), a blood/bone marrow cancer, is a severe and often fatal malignancy. AML cells are capable of impairing the anti-cancer activities of cytotoxic lymphoid cells. This includes the inactivation of natural killer (NK) cells and killing of T lymphocytes. Here we report for the first time that V-domain Ig-containing suppressor of T cell activation (VISTA), a protein expressed by T cells, recognizes galectin-9 secreted by AML cells as a ligand. Importantly, we found that soluble VISTA released by AML cells enhances the effect of galectin-9, most likely by forming multiprotein complexes on the surface of T cells and possibly creating a molecular barrier. These events cause changes in the plasma membrane potential of T cells leading to activation of granzyme B inside cytotoxic T cells, resulting in apoptosis.

## Introduction

Acute myeloid leukemia (AML) is a blood/bone marrow cancer originating from myeloid precursors, which rapidly progresses into a systemic and often a fatal malignancy. Human AML cells operate a variety of biochemical mechanisms which allow them to escape host immune surveillance (1). These molecular pathways cause impairment of the anti-cancer activities of natural killer (NK) cells and cytotoxic T cells which could otherwise attack and kill AML cells (1, 2). It has recently been reported that one of these immune evasion pathways includes high expression/secretion of the protein galectin-9, its receptor and its possible trafficker/carrier (as with all galectins, galectin-9 requires a carrier protein-trafficker to be secreted) - the T cell immunoglobulin and mucin domain containing protein 3 (Tim-3) (3–6). Galectin-9 has two similar ligand/sugar-binding domains (7, 8). These domains are fused together by a peptide linker, which could be of three different sizes (due to alternative splicing), that determines the presence of three isoforms of galectin-9 in human cells (7, 8).

Galectin-9 is actively engaged in impairing the cytotoxic activities of NK cells and can, in addition to suppression, induce apoptotic death of cytotoxic T cells (5, 9–12). However, the mechanisms underlying these differential effects remain unknown. It has been reported that cytotoxic lymphoid cell-based Tim-3 is involved in these effects as a receptor. Tim-3 is expressed by both T cells and NK cells, however, immunosuppressive effects of galectin-9 vary in these cells, suggesting that some additional factors determine the differential responses of NK and T cells to galectin-9 (2, 5). We hypothesized that one of these additional factors could be another immunoglobulin (Ig) superfamily member, known as V-domain Ig-containing suppressor of T cell activation (VISTA), which is expressed in both myeloid and T cells, while NK cells express barely detectable amounts of VISTA (13–15). VISTA has been reported to display both receptor and ligand properties (13–15). Given the similarities between Tim-3 and VISTA structural organizations, we proposed that galectin-9 can interact with T cell-based VISTA causing downstream effects and thus might determine differences in NK and T cell responses to galectin-9.

Here we report for the first time that galectin-9 binds VISTA, most likely as a ligand. Binding was verified using co-immunoprecipitation assays and biophysical methods – synchrotron radiation circular dichroism (SRCD) spectroscopy and surface plasmon resonance (SPR). We confirmed that human T cells, but not NK cells (no VISTA protein was detected by Western blot), express VISTA protein. Both VISTA and Tim-3 mediate galectin-9-induced downregulation of granzyme B (pro-apoptotic protease) release from T cells, increasing the presence of this enzyme inside the T cells which produce it and possibly causing activation of the caspase-3 pro-apoptotic pathway. Furthermore, we found that primary human AML cells secrete high amounts of VISTA compared to healthy mononuclear leukocytes. Exposure of phorbol 12-myristate 13-acetate (PMA – an activator of granzyme B production and release)-activated Jurkat T cells to human galectin-9 and soluble VISTA significantly affects their polarization/membrane potential thus preventing granzyme B release. The same effect was observed in primary human T cells but not in NK cells. We hypothesized that galectin-9 and soluble VISTA can form multiprotein agglomerates engaging with Tim-3 and VISTA on the surface of T cells (but not NK cells which did not show expression of detectable amounts of VISTA protein in Western blot analysis) thus affecting the cell polarity/plasma membrane potential and leading to granzyme B-mediated self-killing.

## Materials and Methods

### Materials

RPMI-1640 cell culture medium, fetal bovine serum and supplements as well as basic laboratory chemicals were purchased from Sigma (Suffolk, UK). Microtitre plates for Enzyme-Linked Immunosorbent Assay (ELISA) were provided by Oxley Hughes Ltd (London, UK). Rabbit antibodies against VISTA, galectin-9, granzyme B and CD3 were purchased from Abcam (Cambridge, UK). Goat antibody against VISTA was purchased from Santa Cruz Biotechnology (Heidelberg, Germany). Antibodies against actin were purchased from Abcam (Cambridge, UK) and Proteintech (Manchester, UK). Mouse antibody against PARP was obtained from Enzo Life Sciences (Exeter, UK). Goat anti-mouse, anti-rabbit and donkey anti-goat fluorescence dye-labeled antibodies were obtained from Li-COR (Lincoln, Nebraska USA). ELISA-based assay kits for detection of galectin-9, Tim-3 and VISTA as well as human recombinant galectin-9 and anti-VISTA antibody reacting to native protein were purchased from Bio-Techne (R&D Systems, Abingdon, UK). VISTA-Fc and Fc human recombinant proteins were obtained from Sino Biological US Inc (Wayne, PA, USA). Anti-Tim-3 mouse monoclonal antibodies (detection and neutralizing) as well as human Ig-like V-type domain of Tim-3 (amino acid residues 22-124), expressed and purified from *E. coli* (16) were used in our work. Antibodies for fluorescent microscopy and flow cytometry as well as annexin V/propidium iodide apoptosis assay kits were from Invitrogen (Carlsbad, USA). All other chemicals purchased were of the highest grade of purity commercially available.

### Cell Lines and Primary Human Samples

THP-1 human myeloid leukemia monocytes, Jurkat T cells and MCF-7 human breast cancer cells were obtained from the European Collection of Cell Cultures (Salisbury, UK). HaCaT keratinocytes were purchased from CLS (Cell Line Service, Germany) and cultured according to the CLS recommendations.

Blood plasma of healthy human donors was obtained as described (17) from buffy coat blood (purchased from healthy donors undergoing routine blood donation) which was purchased from the National Health Blood and Transfusion Service (NHSBT, UK) following ethical approval (REC reference: 16-SS-033). Mononuclear-rich leukocytes were isolated using Ficoll-density centrifugation according to the manufacturer’s protocol. Cell numbers were determined using haemocytometers and then diluted with HEPES-buffered Tyrode’s solution before treatment as indicated in the text. NK cells were purified as previously described (5). Primary human T cells were purified using a commercial T cell purification kit (EasySep Human T Cell Isolation Kit, StemCell Technologies, Cologne, Germany). Primary human AML plasma samples and cells obtained from newly diagnosed AML patients were provided by the sample bank of University Medical Centre Hamburg-Eppendorf (Ethik-Kommission der Ärztekammer Hamburg, reference: PV3469). Cells were kept in IMDM medium containing 15% BIT 9500 serum substitute, 100 µM mercaptoethanol, 100 ng/ml stem cell factor (SCF), 50 ng/ml FLT3, 20 ng/ml G-CSF, 20 ng/ml IL-3, 1 µM UM729 and 500 nM stemregenin 1 (SR1).

### Western Blot Analysis

VISTA, Tim-3, PARP cleavage and CD3 levels were analyzed by Western blot and compared to the amounts of β-actin (protein loading control), as previously described (18). Briefly, cells were lysed in using the buffer (50 mM Tris–HCl, 5 mM EDTA, 150 mM NaCl, 0.5% Nonidet-40, 1 mM PMSF, pH 8.0). After centrifugation, protein content in supernatants was analyzed using Bradford assay. Proteins were resolved using SDS–polyacrylamide gels followed by blotting onto nitrocellulose membranes. Molecular weights were calibrated in proportion to the running distance of rainbow markers. All primary antibodies were diluted 1:1000. Li-COR goat secondary antibodies (dilution 1:2000), conjugated with fluorescent dyes, were used in accordance with manufacturer’s protocol to visualize target proteins (using a Li-COR Odyssey imaging system). Western blot data were analyzed using Odyssey software and values were subsequently normalized against those of β-actin (loading control).

### *In Vitro* Assay of VISTA-Galectin-9 Interactions

This assay was performed as described before for Tim-3-galectin-9 interactions. Briefly, VISTA protein from Jurkat T cell lysates was first precipitated on Maxisorp ELISA plates. For this purpose ELISA plates were coated overnight with goat antibody against VISTA. Plates were then blocked with 2% BSA. Tissue culture medium obtained from culturing PMA-treated THP-1 or MCF 7 (negative control) cells was then applied for 2 h at room temperature, followed by extensive washing with TBST buffer. Proteins were then extracted using 0.2 M glycine-HCl buffer (pH 2.0). Extracts were neutralized using lysis buffer and subjected to Western blot analysis (samples where not boiled in this case) using rabbit anti-galectin-9 and mouse anti-Tim-3 antibodies as described before (4) and above. Alternatively, the format was subjected to measurement of bound galectin-9 using an ELISA kit according to the manufacturer’s protocol.

### Enzyme-Linked Immunosorbent Assays (ELISAs)

Secreted galectin-9 and soluble VISTA, were measured either in cell culture medium or in blood plasma by ELISA using R&D Systems kits according to manufacturer’s protocols.

### On-Cell Western Analysis

We employed LI-COR on-cell Western (OCW) assay to analyze total Tim-3 and VISTA expressions in the studied cells using the Li-COR Odyssey imaging system as previously described (4, 19).

### Fluorescent Microscopy and Flow Cytometry

Cells were cultured overnight on 12 mm cover slips in 24-well plates and then fixed/permeabilised for 20 min with ice-cold MeOH/acetone (1:1). Alternatively cells were fixed in a freshly prepared 2% paraformaldehyde, washed three times with PBS and then permeabilised with 0.1% TX-100. Cover slips were blocked for 1h at RT with 10% goat serum in PBS. Cells were stained with anti-VISTA antibody overnight at 4°C. As secondary antibodies were used goat-anti-mouse Alexa Fluor 488 for 45 min at RT. The nuclei were stained with 4′,6-diamidino-2-phenylindole (DAPI). The preparations were analyzed using Olympus microscope as described previously (16, 20). Images were collected and analyzed using proprietary image acquisition software.

Flow cytometry experiments were performed in accordance with a previously described protocol (21). Briefly, cells were collected and fixed with 2% paraformaldehyde and permeabilised with 0.1% TX-100. Cells were stained with mouse anti-VISTA antibodies conjugated with Alexa Fluor 488 o/n at 4°C. Mean fluorescence intensity of stained cells was measured and analyzed using a FACSCalibur analyzer and the CEllQuest Pro Software (Becton Dickinson, USA).

### Measurement of Granzyme B and Caspase-3 Activities

Granzyme B activity in cell lysates was measured using a fluorometric assay based on the ability of the enzyme to cleave the fluorogenic substrate Ac-IEPD-AFC (Sigma). Caspase-3 activity was measured spectrophotometrically based on its ability to cleave its specific substrate Ac-DEVD-pNA. Both assays were performed according to the manufacturers’ protocol. In-cell activity of granzyme B (granzyme B catalytic activity in living cells) was measured by incubation of living cells with 150 µM Ac-IEPD-AFC (granzyme B substrate) for 1 h at 37°C in sterile PBS. This did not affect viability of the cells as measured by MTS assay (Promega kit, measurements were performed according to the manufacturer’s protocol). Total cell fluorescence was then measured in living cells (22) using excitation and emission wavelengths recommended in the Ac-IEPD-AFC manufacturer’s (Sigma) protocol. An equal number of cells, which were not exposed to granzyme B substrate, were used as a control.

### Apoptosis and Cell Viability Assays

The percentage of apoptotic cells was measured using an annexin V/propidium iodide assay kit by flow cytometry according to the manufacturer’s (Invitrogen) instructions. Cell viability was assessed using an MTS assay kit (Promega) according to the manufacturer’s protocol.

### Characterization of Cell Membrane Potential

This was performed using a DiBAC_4_(3) fluorescent probe where the intensity of accumulation in a cell is proportional to its depolarization. This assay was performed as recommended by the manufacturer and described before (23).

### Synchrotron Radiation Circular Dichroism Spectroscopy

Human recombinant VISTA, galectin-9 and Tim-3 as well as VISTA-galectin-9 or VISTA-galectin-9-Tim-3 complexes were characterized using SRCD spectroscopy at beam line 23, Diamond Light Source (Didcot, UK). SRCD measurements were performed with 0.2 µg/ml of samples using a 1 cm path length cell, 3 mm aperture diameter and 40 µl capacity using Module B with a 1 nm increment, 1s integration time, 1.2 nm bandwidth at 23°C (24–26). The obtained results were analyzed with the help of CDApps (25) and OriginPro™.

### Surface Plasmon Resonance

This assay was performed using a CM5 sensor chip. A Biacore amino coupling kit was employed to immobilize galectin-9 on the chip surface and VISTA (as a fusion protein with Fc) as well as Fc alone were flowed through in order to assess interactions using a Biacore T200 instrument. Data analysis was performed using Biacore T200 software and also using exponential decay (GraphPad Prism), taking into account both association and dissociation (27).

### Statistical Analysis

Each experiment was performed at least three times and statistical analysis when comparing two events at a time was conducted using a two-tailed Student’s *t*-test. Multiple comparisons were performed by ANOVA. Post-hoc Bonferroni correction was applied. Statistical probabilities (p) were expressed as * when p<0.05; **, p<0.01 and *** when p<0.001.

## Results

### Galectin-9 Specifically Binds VISTA With High Affinity

**Figure 1:**
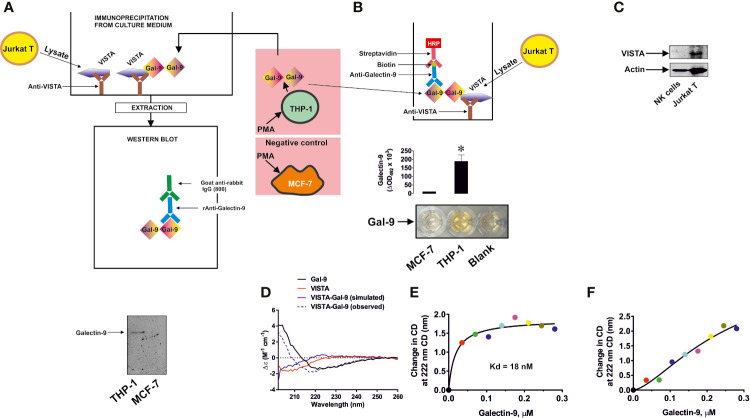
Specific interaction of VISTA with galectin-9. VISTA was captured on the enzyme-linked immunosorbent assays (ELISA) plate using goat anti-VISTA antibody. It was then exposed to medium used to culture phorbol 12-myristate 13-acetate (PMA)-pre-treated (24 h with 100 nM PMA) THP-1 acute myeloid leukemia (AML) cells, secreting galectin-9, or MCF-7 breast cancer cells which do not secrete this protein. Bound proteins were then extracted and subjected to Western blot analysis of galectin-9 **(A)**. Alternatively, the format was subjected to ELISA-based detection of galectin-9 **(B)**. VISTA protein expression was measured using Western blot analysis in primary human NK cells and Jurkat T cells **(C)**. Binding of human recombinant VISTA (used as a fusion protein with human Ig-Fc) and galectin-9 was analyzed using synchrotron radiation circular dichroism (SRCD) spectroscopy. The spectrum of human Ig-Fc was subtracted from the fusion protein VISTA-Fc **(D)**. Binding affinity of galectin-9 to VISTA was analyzed using SRCD titration using a fixed concentration of VISTA-Fc and increasing concentrations of galectin-9 **(E)**. The same experiment was performed using human Ig-Fc instead of VISTA-Fc **(F)**. Images are from one experiment representative of five which gave similar results. Data are shown as mean values ± SEM of five individual experiments. SRCD experiments were performed six times each and average results are presented; *p < 0.05 *vs* control.

In order to assess the possible interactions of VISTA with galectin-9 we used 96 well ELISA plates coated with goat anti-VISTA antibody. We then immunoprecipitated VISTA from Jurkat T cells by exposing the plate to Jurkat T cell lysates (**Figures 1A, B**) as previously described (4). We found that these cells express VISTA protein, while we could not detect any VISTA in primary NK cell lysates (**Figure 1C**; a full comparison of fluorescence observed for VISTA in Western blot analysis conducted in Jurkat T, primary human NK, THP-1, primary human AML and primary human T cells is presented in **Supplementary Figure 1**). When VISTA was captured and attached to an ELISA plate surface through the antibody, we exposed this di-protein complex to medium obtained from culturing THP-1 human AML cells for 24 h in the presence of 100 nM PMA (in order to maximize galectin-9 secretion (5)), which triggered the cells to produce around 6 ng of galectin-9 per 10^6^ cells (as measured by ELISA). As a negative control we used medium obtained from culturing MCF-7 breast cancer cells for 24 h in the presence of 100 nM PMA. MCF-7 cells express but don’t secrete any galectin-9 (6) that was also confirmed here by ELISA where no release of galectine-9 was detected)). After 2 h of exposure we either eluted bound proteins using glycine-HCl buffer (pH 2.0) and subjected the extract to Western blot analysis (4) (**Figure 1A**) or assessed galectin-9 concentrations by ELISA (**Figure 1B**). Western blot analysis demonstrated that galectin-9 was present in samples exposed to the medium from THP-1 but not MCF-7 cells (**Figure 1A**). ELISA analysis also confirmed the presence of galectin-9 in samples exposed to the medium from THP-1 but not MCF-7 cells (**Figure 1B**). Importantly, exposure of the ELISA plate coated with anti-VISTA antibody (and BSA-blocked) to galectin-9 containing medium did not result in any signal, also confirming specific interaction of galectin-9 with VISTA (data not shown). SRCD spectroscopy showed that human recombinant galectin-9 binds VISTA causing conformational changes (**Figure 1D**) and titration showed that the Kd of this binding is 18 nM which confirms a relatively high binding affinity (**Figure 1E**). Since VISTA was used as an immunoglobulin fragment crystallisable region (VISTA-Fc) fusion protein, we checked if Fc can bind galectin-9, but the result was negative, suggesting that there is no specific interaction between these two proteins (**Figure 1F**). For the experiment shown in **Figure 1D** the observed spectra of Fc protein were subtracted from those of VISTA-Fc.

In order to further confirm specific interactions between galectin-9 and VISTA, we performed SPR analysis with galectin-9 immobilized on a CM5 Biacore sensor chip with either VISTA-Fc or Fc flowing through the cell. The binding of VISTA but not Fc was confirmed and we used three different approaches to calculate the Kd – a Lineweaver–Burk type plot, Biacore T200 software and GraphPad prism (exponential decay, association and dissociation approach), see **Supplementary Figure 2**. The Kd in all cases was approximately 100 nM (specific values for each method are shown in **Supplementary Figure 2**). These results confirmed specific binding of galectin-9 to VISTA.

### VISTA Mediates Galectin-9-Induced Suppression of Granzyme B Release and Pro-Apoptotic Processes in T Cells

**Figure 2:**
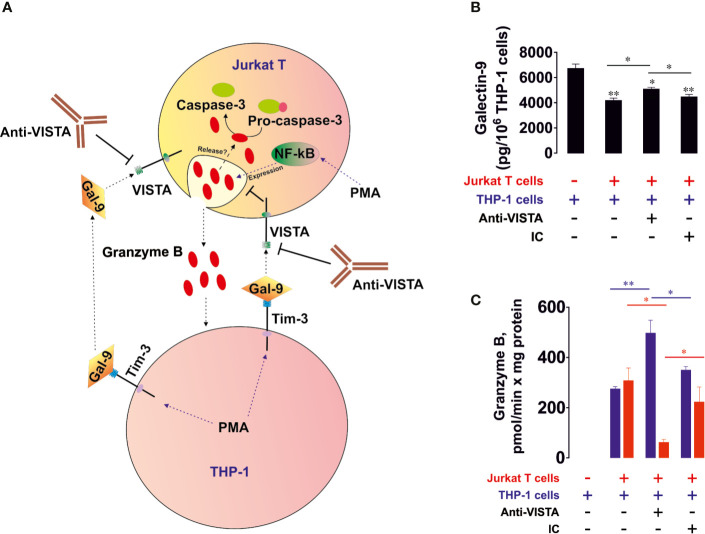
VISTA prevents the release of granzyme B from human T cells into acute myeloid leukemia (AML) cells. THP-1 human AML cells and Jurkat T cells were pre-treated 24 h with 100 nM phorbol 12-myristate 13-acetate (PMA). Medium was changed and the cells were co-cultured at a ratio of 1:1 for 16 h in the absence or presence of VISTA-neutralizing antibody or isotype control antibody **(A)**. Levels of secreted galectin-9 were measured by enzyme-linked immunosorbent assays (ELISA) **(B)**. Granzyme B activity was measured in both THP-1 and Jurkat T cells as outlined in *Materials in Methods* **(C)**. Data are shown as mean values ± SEM of four independent experiments; *p < 0.05, **p < 0.01 *vs* control or indicated events.

To assess the possible role of granzyme B, a pro-apoptotic proteolytic enzyme produced mainly by cytotoxic T cells and NK cells, in galectin-9-induced killing of T cells, we co-cultured THP-1 human AML cells and Jurkat T cells (at a ratio 1:1; Jurkat T cells express high amounts of VISTA and can express granzyme B and release this enzyme upon stimulation with PMA (28, 29), which activates granzyme B expression through NF-kB (29)). Both cell types were pre-treated for 24 h with 100 nM PMA before being co-cultured in PMA-free medium. Induction of granzyme B expression by PMA in Jurkat T cells was confirmed by Western blot analysis (**Supplementary Figure 3**). This led to the adhesion of THP-1 cells and caused them to release high levels of galectin-9 (>6 ng per 10^6^ cells over 16 h of incubation in PMA-free medium after pre-treatment) and activation of granzyme B in Jurkat T cells (these cells did not adhere to the surface and remained in suspension) as well as upregulating their capability to release this enzyme. Equal amounts of cells were co-cultured for 16 h (**Figure 2A**) and galectin-9 release (**Figure 2B**) as well as granzyme B activity (**Figure 2C**) were measured in both cell types. Importantly, PMA-pre-treated THP-1 cells released around 250 pg of soluble VISTA per 10^6^ cells during 16 h of post-PMA incubation (as measured by ELISA).

We found that PMA-treated THP-1 cells produced high levels of galectin-9 which were slightly reduced in the presence of Jurkat T cells (**Figure 2B**), suggesting most likely the interaction of galectin-9 with surface receptors of Jurkat T cells. Importantly, resting Jurkat T cells, pre-treated for 24 h with 100 nM PMA, during 16 h of post-PMA incubation in PMA-free medium released *ca.* 300 pg galectin-9 per 10^6^ cells and did not release detectable amounts of soluble VISTA (as measured by ELISA). Resting PMA-pre-treated THP-1 cells did not show detectable granzyme B activity. In the presence of Jurkat T cells granzyme B was almost equally distributed between THP-1 and Jurkat T cells (**Figure 2C**). Importantly, Ac-IEPD-AFC has a similar amino acid sequence to the caspase 8-specific substrate, IETD (often derivative of type Ac-IETD is used). Therefore, caspase 8 has lower affinity to Ac-IEPD-AFC and can thus cleave it. To rule out the involvement of caspase 8 in the effects observed, we measured Ac-IEPD-AFC cleavage in the presence of the 100 µM granzyme B inhibitor benzyloxycarbonyl-ala-ala-asp-chloromethylketone (Z-AAD-CMK) in the reaction mixture. The presence of this inhibitor completely abolished the observed proteolytic activities which allowed us to conclude that the effects seen where caused by granzyme B and not by caspase 8 or other proteolytic enzymes (**Supplementary Figure 4**). When the co-culture was performed in the presence of 5 μg/ml anti-VISTA neutralizing antibody (R&D Systems), granzyme B activity was mainly detectable in Jurkat T cells. The presence of isotype control antibody didn’t cause any changes compared to co-culture in the absence of antibodies. The differences in effects were also observed in THP-1 cells (**Supplementary Figure 5A**). Importantly, granzyme B in T cells is located in acidic granules, where it doesn’t display catalytic activity since this requires neutral (cytosolic) pH (30). By lysing the cells, granzyme B is co-extracted from the granules, thus enabling the measurement of its total activity. To confirm that granzyme B displays increased activity inside living Jurkat T cells in the presence of THP-1 cells, we measured in-cell activity of the enzyme in resting PMA (100 nM, 24 h) pre-treated Jurkat T cells after 16 h of incubation in PMA free medium and in Jurkat T cells (also pre-treated 24 h with 100 nM PMA) co-cultured for 16 h with PMA-pre-treated THP-1 cells. Then Jurkat T cells (in suspension) were separated from THP-1 cells (adherent) and incubated for 1 h at 37°C in the presence of 150 µM Ac-IEPD-AFC (granzyme B substrate). Cells were then precipitated and fluorescence was measured against equal amounts of the same cells not exposed to the substrate. We found that in-cell activity of granzyme B was significantly increased in Jurkat T cells co-cultured with THP-1 cells (**Supplementary Figure 5B**).

Our observations suggest that VISTA protein is involved in galectin-9-induced suppression of granzyme B release from T cells. Importantly, both VISTA and Tim-3 neutralizing antibodies used in this study were tested and it was found that they blocked the effects of the target proteins, but did not induce any of the downstream effects of their target proteins suggesting no agonistic properties (data not shown). When recombinant Tim-3 or VISTA were immobilized on the surface of the ELISA plate and exposed to galectin-9 for 2 h, galectin-9 was detectable using ELISA detection antibody. When, before exposure to galectin-9, immobilized proteins were pre-incubated with corresponding neutralizing antibodies, galectin-9 was no longer detectable. Neither of the antibodies induced Tim-3 signaling reported earlier for agonistic anti-Tim-3 antibody (16) or pro-apoptotic effects in Jurkat T cells expressing VISTA.

**Figure 3:**
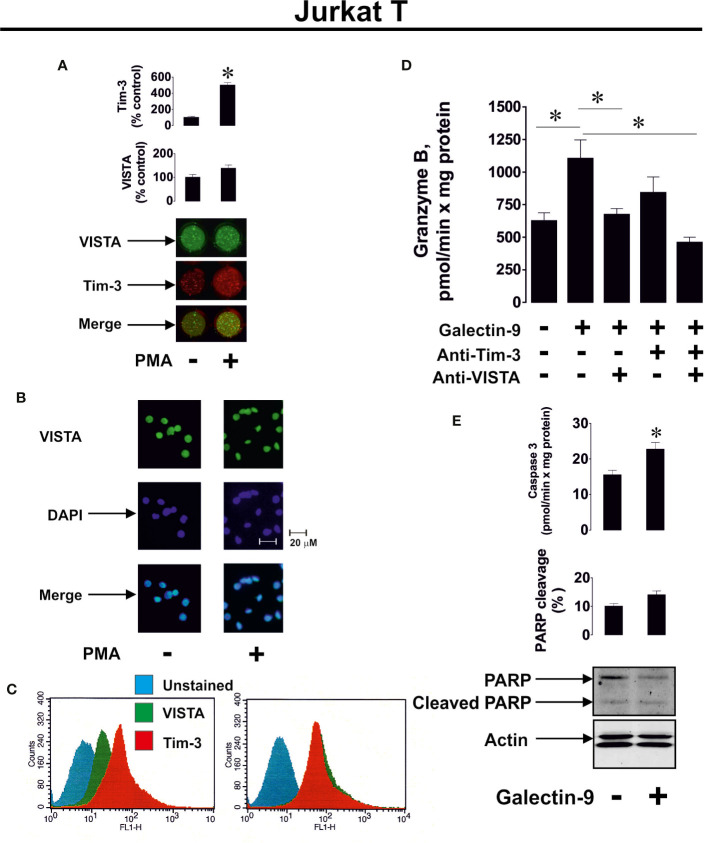
Galectin-9 increases the intracellular activity of granzyme B and upregulates pro-apoptotic processes in Jurkat T cells in a VISTA- and Tim-3-dependent manner. VISTA and Tim-3 expression on the surface of resting Jurkat T cells and those treated for 24 h with 100 nM PMA was measured using anon-cell Western **(A)**. VISTA surface expression was confirmed using immunofluorescence microscopy **(B)**. Total cellular levels of Tim-3 and VISTA were measured in permeabilised Jurkat T cells using flow cytometry **(C)**. Following pre-treatment of Jurkat T cells for 24 h with 100 nM PMA, they were exposed for 24 h to 2.5 µg/ml galectin-9 in the absence or presence of VISTA or/and Tim-3 neutralizing antibodies. Granzyme B activity was then measured in these cells **(D)**. To assess pro-apoptotic processes Jurkat T cells were pre-treated for 24 h with 100 nM PMA and then exposed for 24 h to 2.5 µg/ml galectin-9. Caspase-3 activity and PARP cleavage were then measured by colorimetric assay and Western blot, respectively **(E)**.

We then verified whether human recombinant galectin-9 causes the same effects in PMA-activated Jurkat T cells. We found that VISTA was present on the surface of Jurkat T cells regardless of PMA treatment, while Tim-3 mainly appeared on the cell surface after 24 h of exposure to 100 nM PMA (**Figure 3A**, importantly, expression levels of Tim-3 in Jurkat T cells are lower compared to AML cells, for example THP-1). The presence of VISTA was confirmed using immunofluorescent microscopy (**Figure 3B**) and the total amounts of both VISTA and Tim-3 were analyzed using FACS (**Figure 3C**). Exposure of Jurkat T cells (following 24 h pre-treatment with 100 nM PMA) to 2.5 μg/ml galectin-9 induced the activation of cell-associated granzyme B and the effect was downregulated when cells were pre-exposed (before treatment with galectin-9) to 5 μg/ml Tim-3-neutralizing or VISTA-neutralizing antibody. The presence of both antibodies attenuated the galectin-9-induced effect (**Figure 3D**). Importantly, this experiment confirmed the role of Jurkat T-associated VISTA/Tim-3 in galectin-9-induced effects (in this experiment soluble VISTA/Tim-3 were not present in the medium but they were present on the surface of Jurkat T cells).

To investigate if galectin-9 induces pro-apoptotic effects in PMA-activated Jurkat T cells, we exposed them to 2.5 μg/ml galectin-9 for 16 h and measured caspase-3 activity (colorimetric assay) as well as poly-ADP-ribose polymerase (PARP) cleavage (Western blot). We found that caspase-3 activity was significantly upregulated by galectin-9 and this resulted in a tendency for increased PARP cleavage (**Figure 3E**).

**Figure 4:**
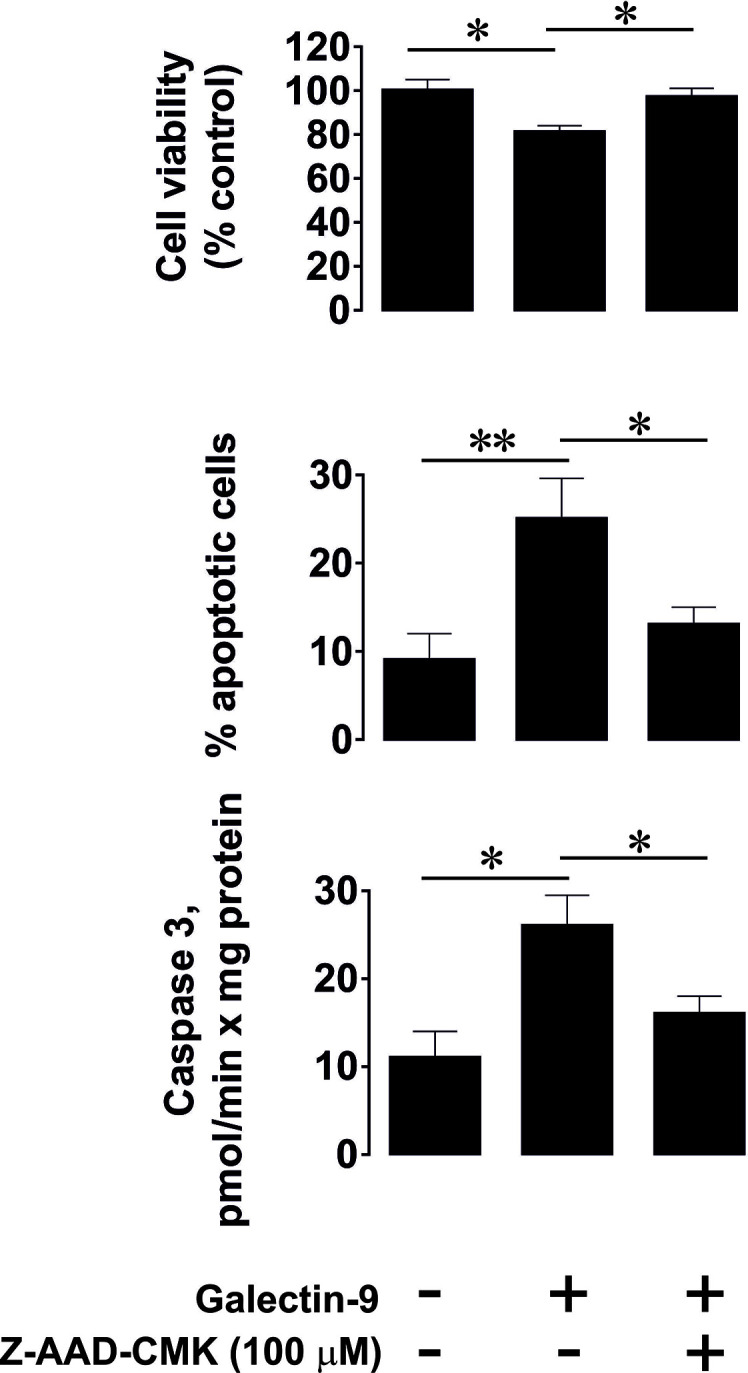
Granzyme B is involved in galectin-9-induced caspase-3 activation and apoptotic death of phorbol 12-myristate 13-acetate (PMA)-pre-treated Jurkat T cells. Jurkat T cells were pre-treated with 100 nM PMA for 24 h followed by 24 h exposure to 2.5 µg/ml galectin-9 with or without 30 min pre-treatment with 100 µM of the granzyme B inhibitor Z-AAD-CMK. The percentage of apoptotic cells was detected using an annexin V/propidium iodide assay kit and pro-apoptotic caspase-3 activity was measured using colorimetric assay. Cell viability was analyzed using an MTS test. Data are shown as mean values ± SEM of three experiments. *p < 0.05, **p < 0.01 *vs* control.

To confirm that caspase-3 activation followed by apoptotic cell death in PMA pre-treated (100 nM, 24 h) Jurkat T cells are granzyme B-dependent, we exposed these cells to 2.5 μg/ml galectin-9 for 24 h with or without 30 min pre-treatment with 100 µM Z-AAD-CMK (granzyme B inhibitor). We then assessed the percentage of apoptotic cells by measuring caspase 3 activity, using a colorimetric assay, in combination with an annexin V/propidium iodide assay. In addition, we measured cell viability using an MTS test. We found that galectin-9-induced caspase-3 activation and apoptotic death were attenuated by the granzyme B inhibitor (**Figure 4**).

**Figure 5:**
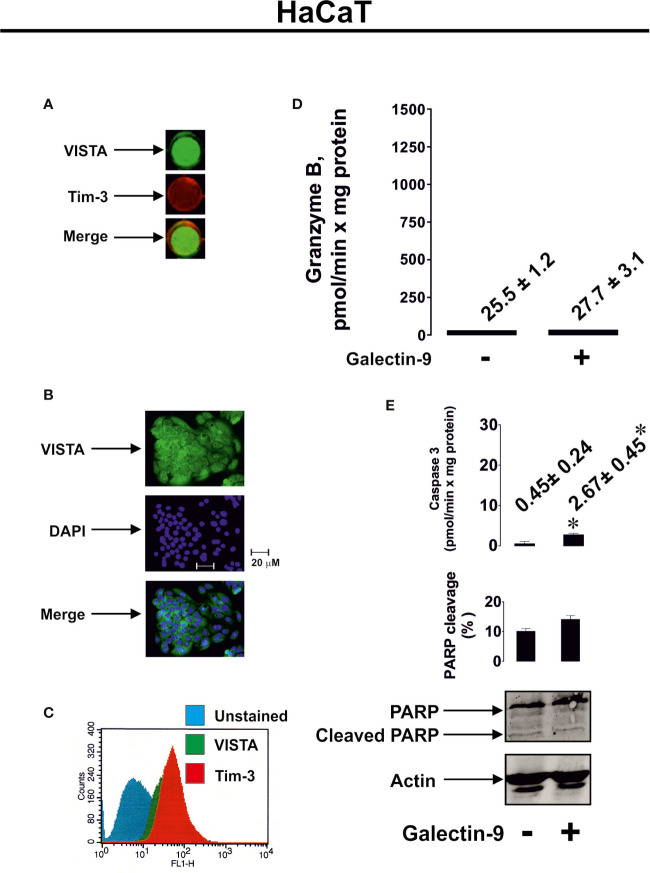
Galectin-9 does not induce pro-apoptotic processes in HaCaT keratinocytes which express VISTA but display almost undetectable granzyme B activity. Expressions of VISTA and Tim-3 were measured on the surface of resting human HaCaT cells (keratinocytes) by on-cell Western **(A)**. VISTA surface expression was confirmed using immunofluorescence microscopy **(B)**. Total cellular levels of Tim-3 and VISTA were measured in permeabilised HaCaT cells using flow cytometry **(C)**. HaCaT cells were exposed for 24 h to 2.5 µg/ml galectin-9 followed by measurement of granzyme B activity **(D)**, caspase-3 activity and poly-ADP-ribose polymerase (PARP) cleavage **(E)**. Images are from one experiment representative of at least four independent experiments which gave similar results. Data are shown as mean values ± SEM for at least four independent experiments; *p < 0.05 *vs* control.

To further confirm the role of granzyme B in galectin-9-induced pro-apoptotic processes we used HaCaT cells (non-malignant human keratinocytes), which express both Tim-3 and VISTA (**Figures 5A–C**), but their granzyme B activity was almost undetectable (**Figure 5D**). Exposure of HaCaT cells to 2.5 μg/ml galectin-9 did not result in any further pro-apoptotic effects (**Figures 5D, E**). However, caspase-3 activity was slightly increased after galectin-9 treatment, but the activity level (only 2.67 ± 0.45 pmol/min per mg protein) was still too low to induce pro-apoptotic processes (approx. 10-fold higher activity is required for this to occur), which is most likely a result of an absence of granzyme B activity in HaCaT cells.

**Figure 6:**
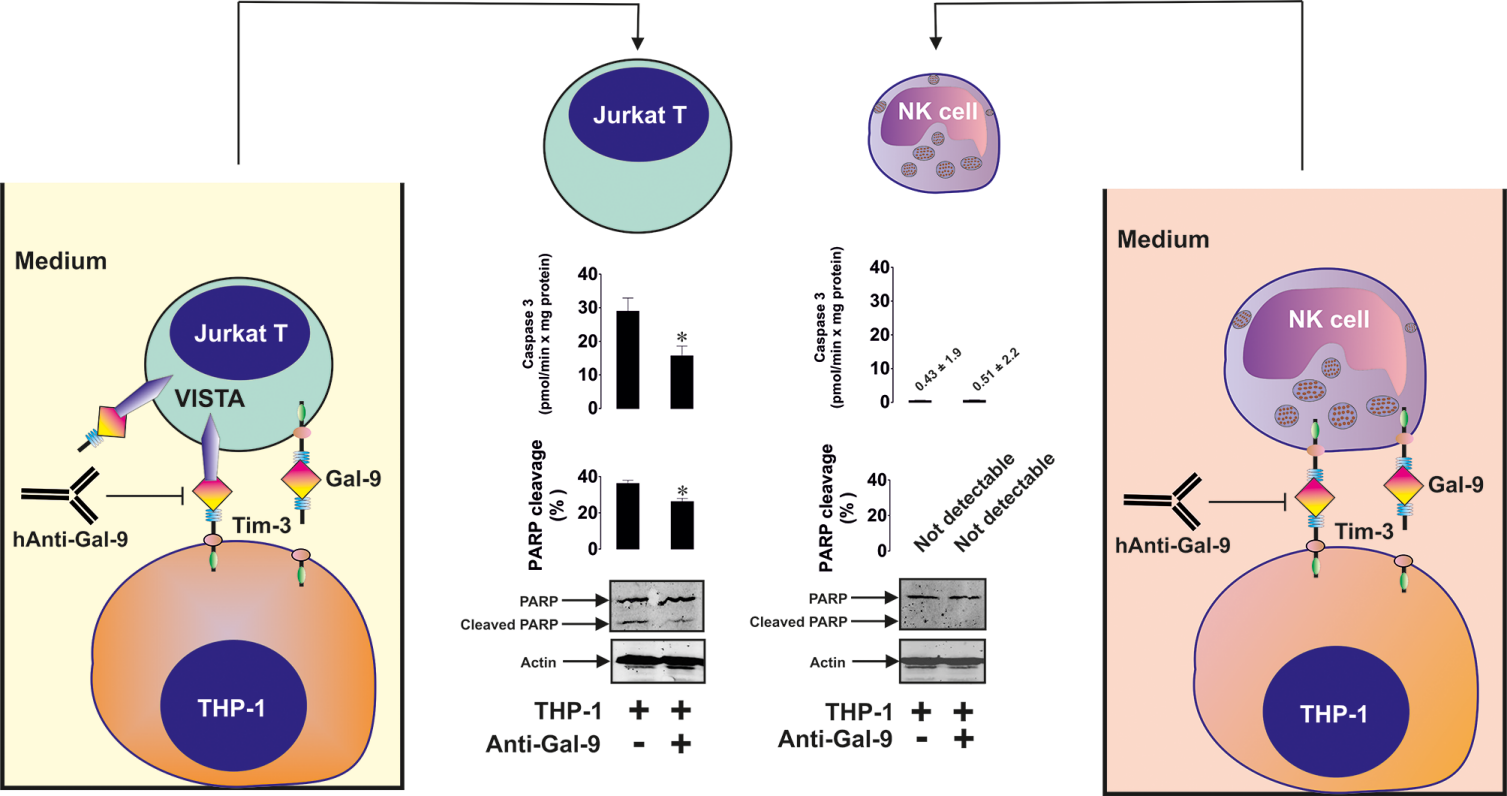
Galectin-9 produced by acute myeloid leukemia (AML) cells induces pro-apoptotic processes in VISTA-expressing T cells but not in VISTA non-expressing NK cells. THP-1 human AML cells were pre-treated for 24 h with 100 nM phorbol 12-myristate 13-acetate (PMA). Then they were co-cultured for 16 h with Jurkat T cells (these cells were also pre-treated with 100 nM PMA for 24 h before co-culturing with THP-1) at a ratio of 1:1or with primary human NK cells at a ratio of 1 THP-1: 2 NK. Caspase-3 activity and PARP cleavage were then measured in Jurkat T and NK cells as outlined in *Materials and Methods*. Images are from one experiment representative of four which gave similar results. Data are shown as mean values ± SEM for four independent experiments; *p < 0.05 *vs* control.

To assess differences in the reactivity of T cells and NK cells to galectin-9 produced by AML cells, we co-cultured 24 h 100 nM PMA-pre-treated THP-1 AML cells with 24 h 100 nM PMA-pre-treated Jurkat T cells (co-culture was performed in PMA-free medium) or primary human NK cells in the absence or presence of anti-galectin-9 antibody for 16 h. In both cases, secreted galectin-9 levels were similar when THP-1 and Jurkat T cells were co-cultured (4.2 ± 0.3 ng/ml) compared to THP-1 cells co-incubated with primary human NK cells (3.9 ± 0.2 ng/ml). We found that the presence of THP-1 cells upregulated PARP cleavage in Jurkat T cells but not in NK cells. This effect was significantly reduced by the presence of anti-galectin-9 antibody in Jurkat T cells (as well as caspase-3 activity) and did not have any influence on the NK cells, which did not produce detectable amounts of VISTA protein (**Figure 6**).

### Soluble VISTA Induces Apoptotic Signaling Cascades in T Cells in Association With Galectin-9

**Figure 7:**
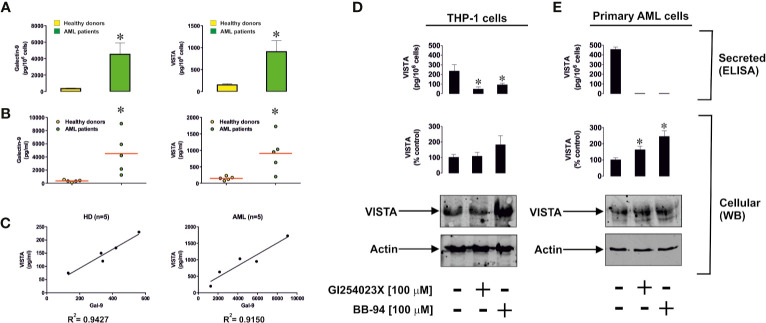
Levels of soluble galectin-9 and VISTA are elevated in the blood plasma of acute myeloid leukemia (AML) patients. **(A)** Blood plasma levels of galectin-9 and VISTA were measured in the blood plasma of five healthy donors and five AML patients. Results are shown as mean values ± SEM of five independent experiments. **(B)** Levels of blood plasma galectin-9 and VISTA in each individual case (average levels are shown by red lines). **(C)** Correlation analysis of galectin-9 and VISTA in blood plasma of healthy donors and AML patients. ADAM10/17 and possibly other matrix metalloproteinases are responsible for proteolytic shedding of VISTA from the surface of phorbol 12-myristate 13-acetate (PMA)-pre-treated (24 h, 100 nM PMA) THP-1 cells **(D)** and primary human AML cells **(E)**. *differences are significant *vs* control – p < 0.05 *vs* healthy donors or cells which were not exposed to protease inhibitors.

Since recent evidence has clearly demonstrated that VISTA protein can be secreted by human myeloid cells, we asked whether human AML cells produce soluble VISTA. We measured VISTA and galectin-9 proteins in the blood plasma of 5 newly diagnosed AML patients and 5 healthy donors. We found that in AML patients, the levels of both proteins were significantly upregulated (**Figures 7A, B**). Furthermore, there was a clear correlation between the levels of both proteins (**Figure 7C**).

We found that isolated primary human healthy leukocytes produce around 220 ± 24 pg of galectin-9 per 10^6^ cells and 89 ± 12 pg of soluble VISTA, while primary human AML cells produced 5980 ± 626 pg of galectin-9 per 10^6^ cells and 707 ± 154 pg of soluble VISTA during 24 h when cultured *in vitro* (**Supplementary Figure 6A**). Using Western blot analysis we determined the molecular weight of soluble VISTA in blood plasma of AML patients, which was observed at 40 kDa (**Supplementary Figure 6B**). This is likely to correspond to the glycosylated extracellular domain of the protein, because deglycosylation performed using a deglycosylation kit (Promega) led to a reduction in molecular weight and the appearance of two new bands at around 36 and 28 kDa (**Supplementary Figure 6B**). Incubation of the samples with deglycosylation enzymes was performed for 3 h in order to observe the deglycosylation path. A longer incubation period (18 h) led to the appearance of a 28 kDa band only (data not shown). Respectively, the bands observed most likely correspond to partially and fully deglycosylated protein. The smallest molecular weight protein is close to that of extracellular protein domain (31). Soluble VISTA is most likely to be produced in a similar manner to soluble Tim-3 and other Ig-type proteins – *via* proteolytic shedding. To confirm this we exposed THP-1 cells (following 24 h pre-treatment with 100 nM PMA) or primary human AML cells to 100 µM GI254023X (an inhibitor of angiotensin and metalloproteinase domain-containing proteins (ADAM) 10/17) or 100 µM batimastat (BB-94, matrix metalloproteinase inhibitor) for 24 h. Given the similarities between Tim-3 and VISTA we hypothesized that they may be shed by the same proteolytic enzymes and thus used these particular protease inhibitors (5). We found that both inhibitors significantly reduced the release of soluble VISTA by THP-1 cells, where the effect of GI254023X was stronger (**Figure 7D**) and attenuated the effect in primary AML cells (**Figure 7E**). These results suggest that, as with Tim-3, VISTA is mainly shed by ADAM10/17 enzymes.

**Figure 8:**
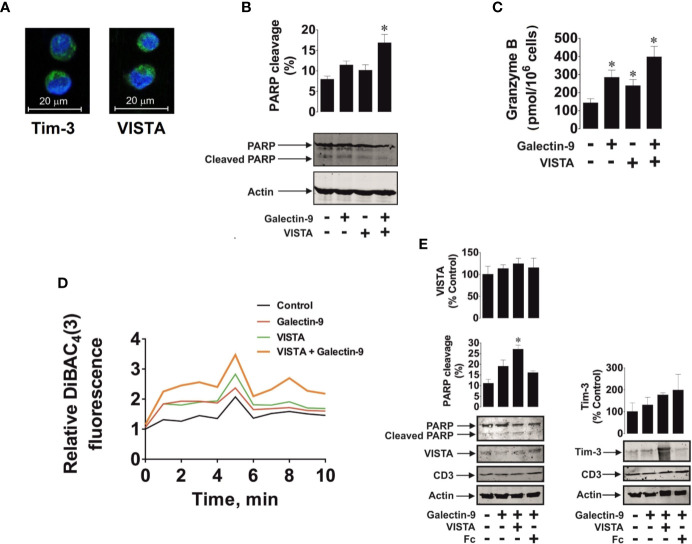
Soluble galectin-9 and VISTA induce pro-apoptotic events in VISTA expressing T cells. Jurkat T cells pre-treated for 24 h with 100 nM PMA or primary human CD3-positive T cells were exposed to human recombinant galectin-9 or/and VISTA. In Jurkat T cells the presence of Tim-3 and VISTA proteins was confirmed using immunofluorescent microscopy **(A)**. Jurkat T cells were exposed to 2.5 µg/ml galectin-9, 5 µg/ml VISTA or combination of both proteins for 16 h. Poly-ADP-ribose polymerase (PARP) cleavage was measured using Western blot analysis **(B)** and in-cell activity of granzyme B – by fluorometric assay **(C)**. Jurkat T cells were exposed for 30 min to 2.5 µg/ml galectin-9, 5 µg/ml VISTA or a combination of both proteins and plasma membrane potential was measured **(D)**. Primary human CD3-positive T cells were exposed for 16 h to 2.5 µg/ml galectin-9, 5 µg/ml VISTA (or Fc to confirm specific effect of VISTA) or combination of both proteins, and PARP cleavage was measured using Western blot analysis. Expression of VISTA, Tim-3, and CD3 was also confirmed using Western blot **(E)**. Images are from one experiment representative of three which gave similar results. Data are mean values ± SEM of three independent experiments; *p < 0.05 *vs* control.

We then exposed 24 h 100 nM PMA pre-treated Jurkat T cells to 2.5 µg/ml galectin-9, 5 µg/ml (to assure use of equimolar amounts of proteins) VISTA or a combination of both proteins in the indicated concentrations for 16 h. Cells clearly expressed both receptors (VISTA and Tim-3) as determined by immunofluorescent microscopy (**Figure 8A**). We then measured PARP cleavage as a marker of caspase-3 induced pro-apoptotic events and found that a combination of galectin-9 and VISTA significantly increased PARP cleavage and obviously reduced the total amount of PARP (**Figure 8B**). In-cell activity of granzyme B was also significantly increased in the presence of galectin-9 or VISTA and was further significantly upregulated in the presence of a combination of both proteins (**Figure 8C**).

To assess if galectin-9 and VISTA could form complexes affecting cell polarity (plasma membrane potential), we exposed 24 h 100 nM PMA pre-treated Jurkat T cells to DiBAC_4_ reactive dye which concentrates in depolarised cells as outlined in *Materials and Methods* and described previously (23). Cells were treated with 2.5 µg/ml galectin-9 or 5 µg/ml VISTA or a combination of both proteins at the indicated concentrations for 30 min. We then measured fluorescence to characterize cell membrane potential over a 10 min period. We found that both galectin-9 and VISTA upregulated fluorescence intensity, however, the effect was substantially greater when cells were exposed to a combination of both proteins (**Figure 8D**). We hypothesized that soluble VISTA and galectin-9 may form multiprotein complexes on the T cell surface which may affect membrane potential and prevent granzyme B release. We sought to confirm whether the three proteins are capable of forming a complex and thus performed SRCD spectroscopy of human recombinant VISTA-Fc (Fc spectra were subtracted), galectin-9 and Tim-3 as well as an equimolar mixture of all three proteins (**Supplementary Figure 7**). There was an obvious interaction of the three proteins since conformational changes were seen in the observed spectrum compared to the simulated spectrum.

We then confirmed the observed effect using primary CD3-positive T cells isolated from the blood of healthy donors. These cells expressed both Tim-3 and VISTA as well as CD3 (a T cell-specific biological marker) as measured by Western blot analysis (**Figure 8E**). Cells were exposed for 16 h to 2.5 µg/ml galectin-9 in the absence or presence of 5 µg/ml VISTA (or 5 µg/ml Fc – the protein fragment to which the recombinant VISTA is fused). We found that, as in PMA-activated Jurkat T cells, a combination of both galectin-9 and VISTA significantly upregulated PARP cleavage, while the presence of galectin-9 alone non-significantly increased it (**Figure 8E**), which is in line with the observations made in Jurkat T cells (**Figures 8B, C**).

Taken together, our results suggest that galectin-9 is a ligand for VISTA, which can induce granzyme B-dependent programmed death of T cells, especially in combination with soluble VISTA. The presence of VISTA in cytotoxic lymphoid cells may determine their responsiveness to galectin-9.

## Discussion

Elucidating the reasons for the differential responsiveness of human NK and T cells to galectin-9 was the main goal of this study. Our results indicate that human T but not NK cells produce detectable amounts of the protein VISTA (**Figures 1C** and **8E**), which can act as immunosuppressive receptor but can also be present in soluble form and function as a ligand. Immunoprecipitation studies and biophysical assays (SRCD spectroscopy and SPR) indicated specific binding of galectin-9 to VISTA with a relatively high affinity (Kd was 18 nM in surface-free SRCD spectroscopy assay and ~ 100 nM when analyzed by SPR assay involving protein immobilization on the surface). Galectin-9 derived from THP-1 human AML cells was shown to downregulate the release of granzyme B (a pro-apoptotic proteolytic enzyme) from PMA-activated Jurkat T cells (**Figure 2**). As shown in **Figure 2**, PMA led to both activation of granzyme B in Jurkat T cells and its release into THP-1 cells. Exposure of these cells to both PMA and calcium ionophore could have led to a higher level of granzyme B activation, as indicated in previous reports (28), but it would also have led to an artificial increase in cytosolic calcium levels. Such an effect could normally lead to artificially enhanced pro-apoptotic effects (32), whereas the aim of our study was to observe more naturally occurring apoptosis.

Our experiments suggested that pro-apoptotic effects can be induced by galectin-9 only in cells which express both VISTA and also produce active granzyme B. This was applicable to PMA-activated Jurkat T cells (**Figures 3** and **4**). Furthermore, galectin-9-induced caspase-3 activation and apoptotic death of these cells were attenuated by 100 µM Z-AAD-CMK (granzyme B inhibitor), suggesting the involvement of granzyme B in the process. The pro-apoptotic effects were not observed in HaCaT keratinocytes which express both Tim-3 and VISTA but granzyme B activity in these cells is barely detectable (**Figure 5**). Primary human NK cells, which express Tim-3, granzyme B but no detectable VISTA, also did not respond pro-apoptotically to galectin-9 released by THP-1 human AML cells (**Figure 6**). Our results suggest a lack of VISTA expression in NK cells and are in line with previously reported observations where flow cytometry tests have shown NK cells, to be mostly negative for VISTA expression (13–15). All these results suggest galectin-9-triggered VITSA/Tim-3-mediated prevention of granzyme B release from T cells. It is interesting to note that AML cell-derived galectin-9 induces the same effects as recombinant protein but in much lower concentrations which suggests that galectin-9 produced by AML cells displays higher activity compared to the recombinant one.

AML cells were found to produce high levels of both proteins – galectin-9 and VISTA (**Figure 7**). In this study we found that both PMA-treated THP-1 and primary human AML cells released soluble forms of galectin-9 and VISTA proteins in high amounts. Blood plasma of newly diagnosed AML patients contains significantly greater levels of soluble VISTA and galectin-9 compared to the blood plasma of healthy donors. There is a clear correlation between the secretion of these two proteins (**Figures 7A–C**). Western blot analysis (before and after deglycosylation) indicated that blood plasma most likely contains the glycosylated extracellular domain of VISTA protein (as in the case of Tim-3, **Supplementary Figure 6B**). Furthermore, we found that VISTA (like Tim-3) is shed from the surface of AML cells by ADAM10/17 proteolytic enzymes (**Figures 7D, E**). Interestingly, simultaneous increases in the expressions of galectin-9, VISTA and Tim-3 were reported for other cancers, for example gastric cancer (33). Our further experiments indicate that soluble VISTA significantly enhances the pro-apoptotic effects of soluble galectin-9 in T cells. This occurs due to changes in cell polarization/membrane potential (**Figure 8D**), which may attenuate the capability of T cells to release granzyme B from the cell. As a result, granzyme B is released from the acidic granules in the cell which produced it and thus displays a high activity within this cell (**Figure 8C**, **Supplementary Figure 5B**). Granzyme B can then induce the classic caspase-3-mediated apoptotic pathway. Furthermore, galectin-9 could possibly upregulate granzyme B expression in T cells in a Tim-3-dependent manner, since presence of the Tim-3-galectin-9 complex on the cell surface leads to activation of NF-kB, which is known to induce granzyme B expression in these cells (29).

We hypothesized that, given the affinity of Tim-3 and VISTA to galectin-9, these three proteins may form agglomerates on the cell surface thereby affecting cell membrane potential/polarization and the ability to release granzyme B. SRCD experiments confirmed that human recombinant VISTA, galectin-9 and Tim-3 can form agglomerates leading to conformational changes of these proteins. Importantly, granzyme B displays highest catalytic activity at neutral pH. Therefore, in cells producing it, this enzyme is stored in acidic granules to prevent its activation (27, 34). However, when T cells such as Tregs (regulatory T cells) and CD8-positive cytotoxic T cells are ready to release granzyme B (34), formation of the multiprotein barrier by galectin-9, VISTA and Tim-3 could potentially lead to its release (leakage) from the granules, thus resulting in its activation within the cells that produce it. In addition, it has been demonstrated that galectin-9 strongly induces calcium mobilization specifically in T cells (35). Unlike T cells, however, this effect can be either very mild or absent in myeloid cells (16, 35). Forming such a barrier around the T cell by galectin-9, VISTA and Tim-3 will most likely preserve a high calcium concentration in the T cells, which can then provoke granzyme B release from the granules and lead to apoptosis induction in granzyme B expressing cytotoxic T cells and Tregs. In helper T cells, where granzyme B activity is barely detectable, an increase in intracellular calcium concentration can lead to calcium-calpain-caspase-dependent apoptosis (32). In support of this assumption, our experiments with HaCaT cells showed a slight increase in caspase-3 activity after exposure to galectin-9 (**Figure 5E**, however the actual caspase-3 activity was substantially lower compared to galectin-9-treated PMA-activated Jurkat T cells), despite granzyme B activity in these cells is almost undetectable. Galectin-9-induced apoptotic effects can be stronger in T helper cells (however, a high concentration of galectin-9 is required (32, 35) than in HaCaT keratinocytes (36), since T cells display strong pro-apoptotic calcium signaling (32, 35).

Serpin B9 is the endogenous inhibitor of granzyme B, which is known to be involved in the protection of cytotoxic lymphoid cells against granzyme B-induced programmed death (37–40). One could hypothesize that if granzyme B is leaking from the intracellular granules of T cells, its concentration is higher than that of serpin B9, similar to the effects observed in NK cells (39). Jurkat T cells, which we studied, almost lack serpin B9 expression (40) and thus we observed proapoptotic events in these cells. In primary T cells, the amount of granzyme B leaking upon exposure to galectin-9 may be higher than those of serpin-9 thus causing pro-apoptotic events. However, this phenomenon requires further investigation in order to understand the detailed intracellular mechanisms leading to galectin-9-induced programmed death of T lymphocytes. Normally diffusion of granzyme B through transmembrane pores appears to be the dominant mechanism of granzyme delivery into the target cells (41, 42). These pores are formed by the pore-forming protein perforin (41, 42) produced by NK and cytotoxic T cells. However, upon changes in plasma membrane potential induced by galectin-9/VISTA, granzyme B release from T cells is most likely affected. Similar effects were described in regulatory T cells (Tregs) (43) and NK cells (39) where granzyme B was shown to leak from cytotoxic granules and induce self-inflicted damage and programmed cell death.

**Figure 9:**
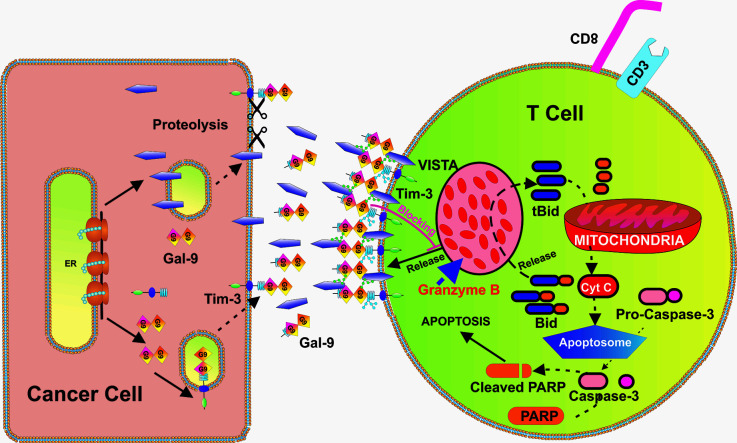
Possible biochemical mechanism underlying VISTA-Galectin-9-Tim-3 induced programmed death of T cells. VISTA, galectin-9 and Tim-3 form a multi-protein complex on the surface of the T cells (glycosides associated with Tim-3 and VISTA further strengthen the interactions). This prevents the release of granzyme B from T cells which express it and results in its activation within the T cells themselves. Activation of granzyme B leads to mitochondrial dysfunction through the Bid pathway that results in cytochrome c release and activation of the caspase-3 apoptotic pathway [which cleaves poly-ADP-ribose polymerase (PARP) and other crucial cellular proteins].

Our results suggest that intracellular leakage of granzyme B may be involved in galectin-9-induced VISTA-dependent cell death too, although other mechanisms, for example calcium-mediated pro-apoptotic events observed when higher galectin-9 concentrations (1 µM or 32 µg/ml) (32), cannot be ruled out. Schematically, our hypothesis of the involvement of granzyme B in VISTA/Tim-3/galectin-9-induced self-killing of T cells is summarized in **Figure 9**.

Taken together, our findings demonstrate for the first time that VISTA interacts with galectin-9 with relatively high affinity, without preventing the interaction of galectin-9 with Tim-3. Soluble VISTA enhances the immunosuppressive activity of galectin-9. Given the affected plasma membrane potential of the cells exposed to a combination of galectin-9 and VISTA, one could suggest that multiprotein complexes are formed by Tim-3, galectin-9 and VISTA (both T cell surface-based and soluble), thus depolarising the cell and forming a barrier which prevents the release of granzyme B. On the other hand, it is possible that soluble VISTA additionally, binds to some other T cells receptors, which remain to be identified, supporting galectin-9-induced pro-apoptotic effects. These effects increase granzyme B activity within the cell that produces it, finally leading to suppressed cellular activity and even to cell death.

## Data Availability Statement

The datasets used and/or analyzed during the current study are available from the corresponding author on reasonable request.

## Ethics Statements

The studies involving human participants were reviewed and approved by Blood plasma of healthy human donors was obtained as described from buffy coat blood (purchased from healthy donors undergoing routine blood donation) which was purchased from the National Health Blood and Transfusion Service (NHSBT, UK) following ethical approval (REC reference: 16-SS-033). Written informed consent for participation was not required for this study in accordance with the national legislation and the institutional requirements.

## Author Contributions

IM, NM, and SS performed majority of the experiments and analysed the data. RH and GS performed SRCD spectroscopy and data analysis. MC-H provided expertise on structural data analysis and compound characterisation. WF and JW isolated and provided primary AML samples used to obtain crucial data. CD and LC helped with performing SPR analysis. LV designed anti-Tim-3 antibodies used in this study. SB and UR participated in design of the concept and planning the experiments as well as writing the manuscript. BG, EF-K, and VS designed the study, planned all the experiments together with IY, analyzed the data, and wrote the manuscript. All authors contributed to the article and approved the submitted version.

## Conflict of Interest

The authors declare that the research was conducted in the absence of any commercial or financial relationships that could be construed as a potential conflict of interest.
