# Supplementary material for: Ligand-Receptor Interactions of Galectin-9 and VISTA Suppress Human T Lymphocyte Cytotoxic Activity
Source: Front Immunol. 2020 Nov 20;11:580557. doi: 10.3389/fimmu.2020.580557 (PMC7715031; doi:10.3389/fimmu.2020.580557)
Supplement: Supplementary file 1 [file DataSheet_1.docx]

**Supplementary information**

**Ligand-Receptor interactions of galectin-9 and VISTA suppress human T lymphocyte cytotoxic activity**

Inna M. Yasinska, N. Helge Meyer, Stephanie Schlichtner, Rohanah Hussain, Giuliano Siligardi, Maxwell Casely-Hayford, Walter Fiedler, Jasmin Wellbrock, Cloe Desmet, Luigi Calzolai, Luca Varani, Steffen M. Berger, Ulrike Raap, Bernhard F. Gibbs, Elizaveta Fasler-Kan and Vadim V. Sumbayev


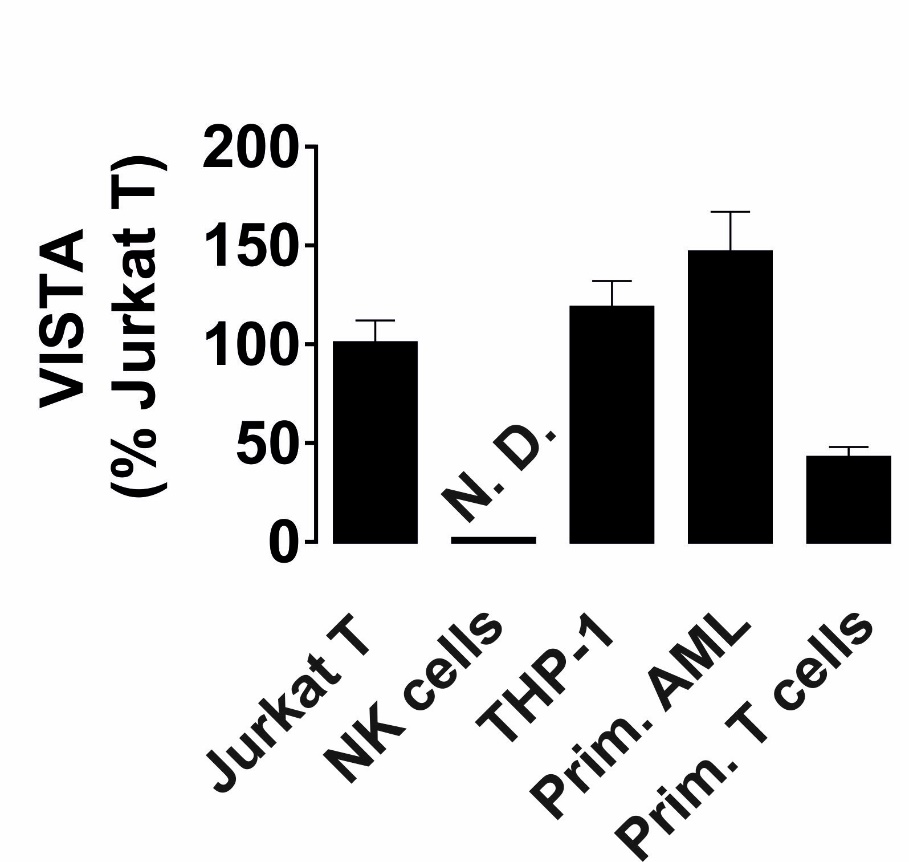


**Supplementary figure 1. Comparative analysis of VISTA expression in various human haematopoietic cell lines and primary cells.** VISTA was measured by Western blot analysis and the observed fluorescence was normalised against total protein loaded onto the gel in each specific case. Data are shown as mean values ± SEM of five independent experiments. In this and other figures N.D. – means “not detectable”


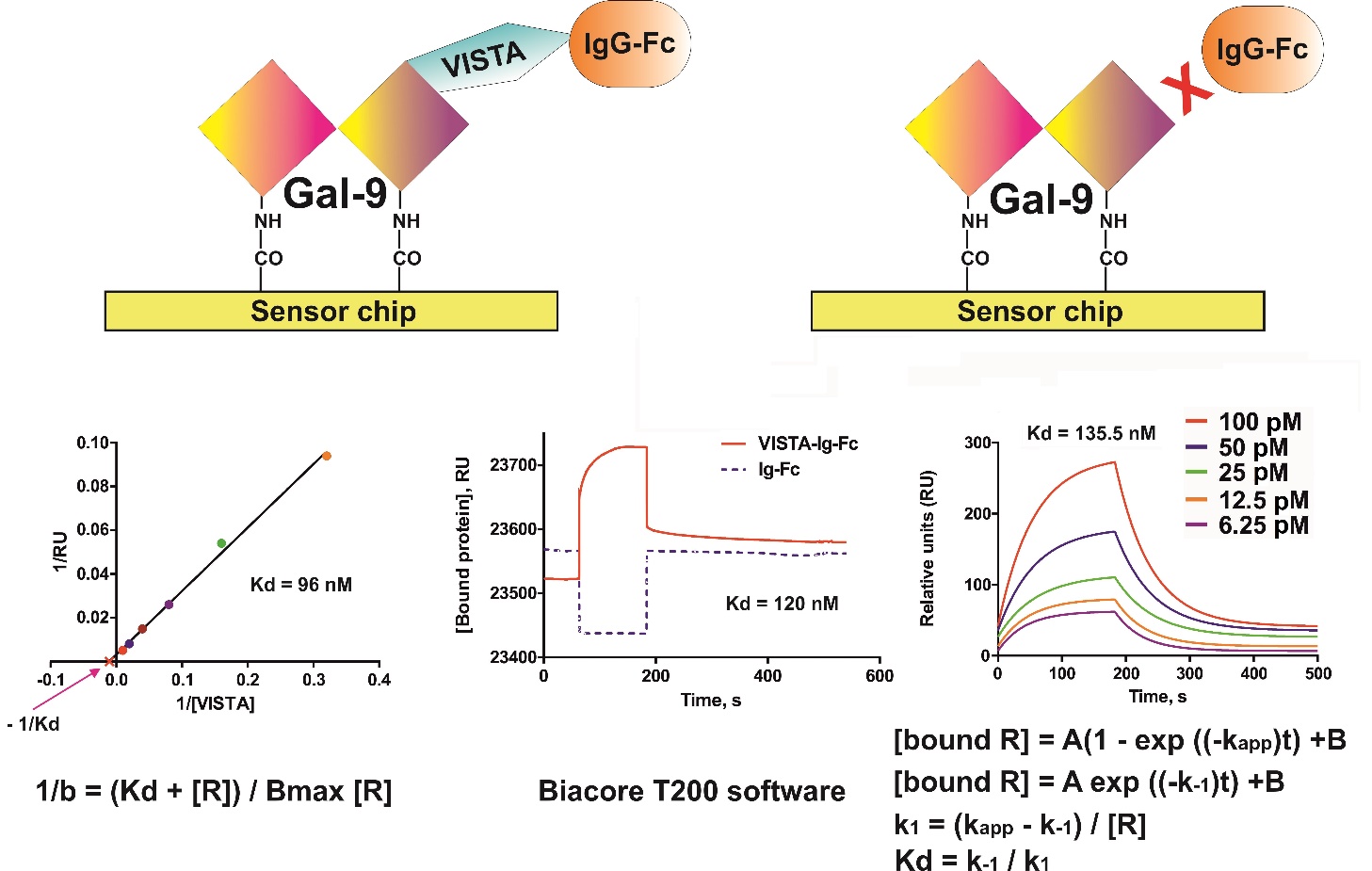


**Supplementary figure 2. Interactions of human galectin-9 and VISTA.** Galectin-9 was immobilised on the CM5 sensor chip using a Biacore amino-coupling kit. VISTA-Fc or Ig-Fc protein was then flowed through to analyse binding. The left diagram shows calculations of the affinity constant using a Lineweaver–Burk plot taking the post-dissociation (bound) levels of galectin-9 into account. The middle diagram shows curves obtained with 100 pM VISTA-Fc and 100 pM Ig-Fc and the Kd calculated using Biacore T200 software. The right diagram shows fitted curves obtained using increasing concentrations of VISTA-Fc (6.25, 12.5, 25, 50 and 100 pM VISTA-Fc). Before fitting the curves the values, obtained when flowing Fc, were subtracted. Curve fitting and Kd calculations were performed using exponential decay, taking into account both association and dissociation performed in GraphPad Prism.


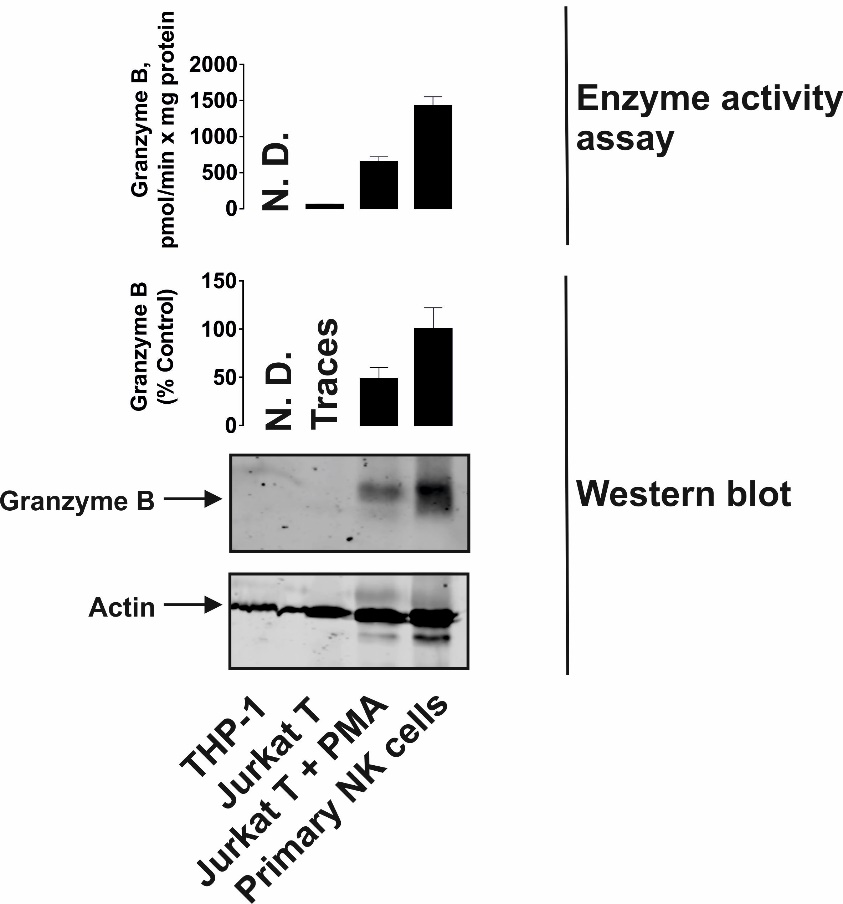


**Supplementary Figure 3. Granzyme B protein levels and proteolytic activity in studied human cells.** Granzyme B protein levels were measured by Western blot analysis in THP-1 cells, resting Jurkat T cells, Jurkat T cells pre-treated 24 h with 100 nM PMA and primary human NK cells (positive control). Catalytic activity of granzyme B was measured as outlined in Materials and Methods. Images are from one experiment representative of four which gave similar results. Data are the mean values ± SEM of four independent experiments.


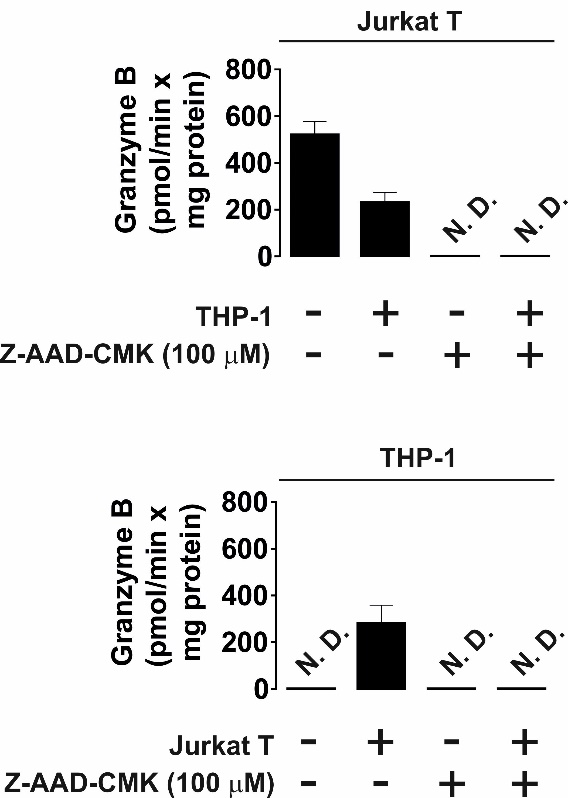


**Supplementary figure 4. Ac-IEPD-AFC cleaving activities observed in Jurkat T and THP-1 cells are specific to granzyme B.** THP-1 human AML cells and Jurkat T cells were pre-treated 24 h with 100 nM PMA. Medium was changed and the cells were co-cultured at a ratio of 1:1 for 16 in the absence or presence of 100 µM granzyme B inhibitor benzyloxycarbonyl-ala-ala-asp-chloromethylketone (Z-AAD_CMK). Granzyme B activity was measured as outlined in Materials and Methods. Data are shown as mean values ± SEM of four independent experiments.


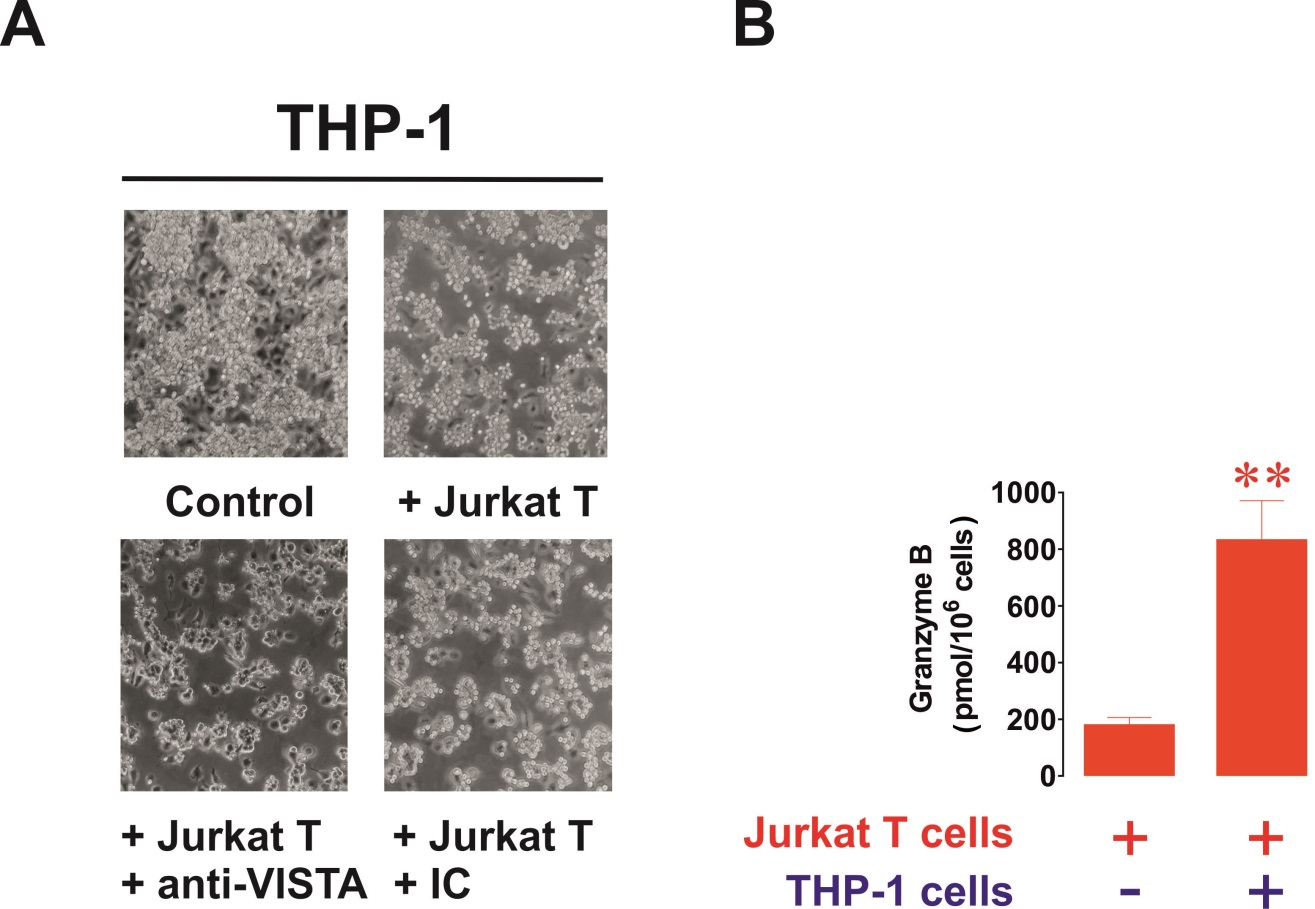


**Supplementary figure 5. AML cell-derived galectin-9 affects T cells by upregulation of their granzyme B activity in a VISTA-dependent manner.** THP-1 human AML cells and Jurkat T (human T cells) cells were pre-treated 24 h with 100 nM PMA. Cells were co-cultured at a ratio of 1:1 for 16 h in PMA-free medium in the absence of presence of VISTA-neutralising or isotype control antibody. (**A**) THP-1 cells were observed after being exposed to activated Jurkat T cells. (**B**) In-cell granzyme B activity was measured in resting PMA-activated Jurkat T cells and those co-cultured for 16 h with THP-1 cells at a ratio of 1:1. Images are from one experiment representative of four, which gave similar results. Data are shown as mean values ± SEM of four experiments. ** - p < 0.01 *vs* control.


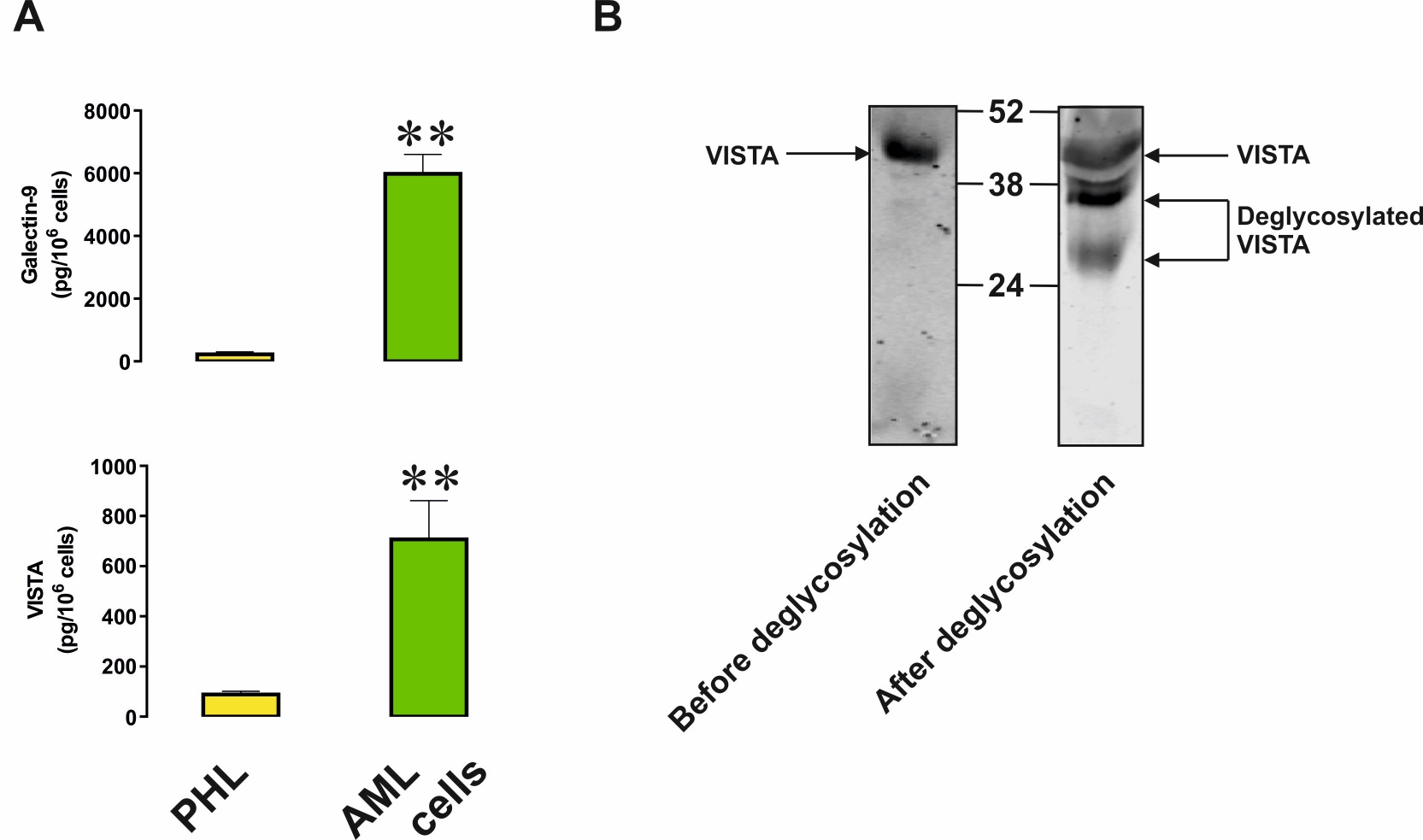


**Supplementary figure 6. Primary human AML cells release high levels of soluble VISTA.** (**A**) Primary human healthy leukocytes (PHL) and AML cells were cultured for 24 h and the levels of secreted galectin-9 and VISTA were measured by ELISA. (**B**) The presence of VISTA (unmodified and following deglycosylation) was analysed in the blood plasma of AML patients using Western blot analysis. Images are from one experiment of five, which gave similar results. Data are shown as mean values ± SEM of five independent experiments. ** - p < 0.01 *vs* PHL.


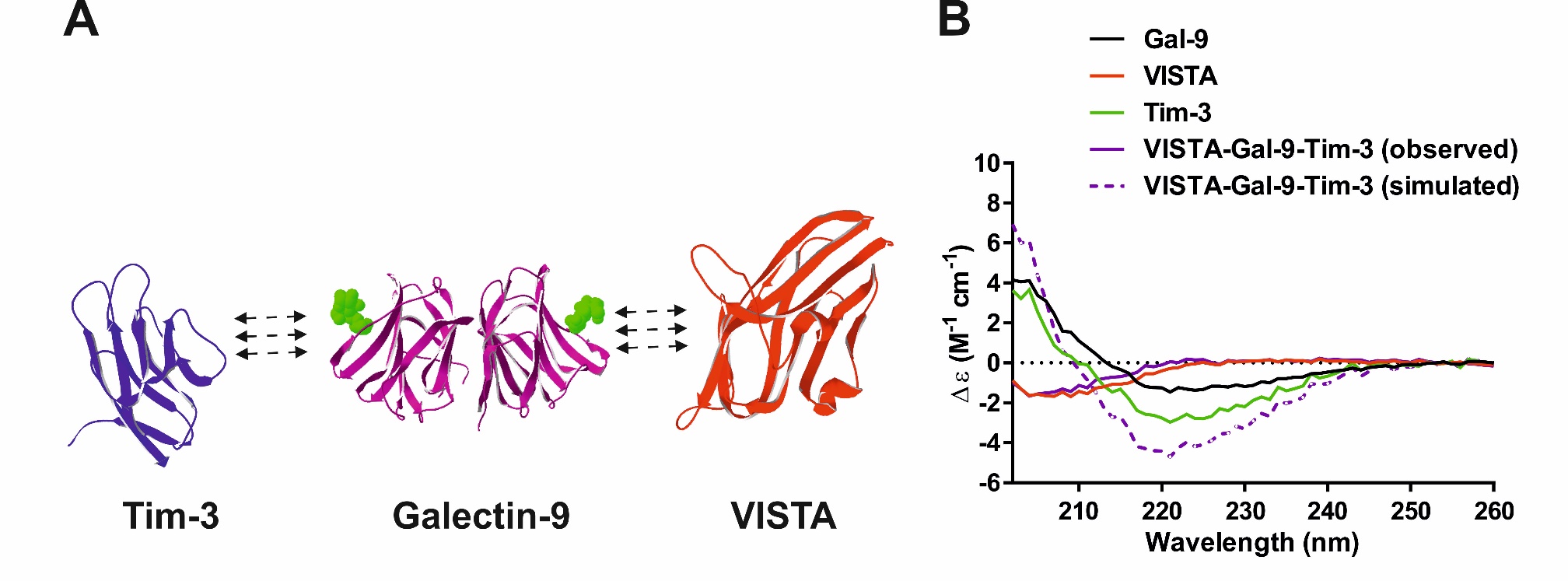


**Supplementary figure 7. Interactions between human recombinant Galectin-9, VISTA and Tim-3.** (**A**) 3D structures of Tim-3, galectin-9 and VISTA were constructed using a Swiss PDB viewer. (**B**) Analysis of the SRCD spectra of Galectin-9, Tim-3 and VISTA-Fc (Ig-Fc spectrum was subtracted) and the interaction of these proteins mixed in equimolar amounts. The observed curves represent an average of six similar curves.
